# Toward more intuitive prosthetic control: structured residual muscle training in transtibial amputees

**DOI:** 10.1186/s12984-026-01989-6

**Published:** 2026-04-16

**Authors:** Faranak Rostamjoud, Friðrika Björk Þorkelsdóttir, Atli Örn Sverrisson, Sigurður Brynjólfsson, Kristín Briem

**Affiliations:** 1 School of Health Sciences, University of Iceland, 101 Reykjavík, Iceland; 2 Össur ehf., 110 Reykjavík, Iceland; 3 School of Engineering and Natural Sciences, University of Iceland, 101 Reykjavík, Iceland

**Keywords:** Biofeedback training, Co-contraction, EMG, Muscle activation, Myoelectric control, Rehabilitation

## Abstract

**Background:**

Myoelectric control of lower-limb prostheses is challenging due to residual muscle co-contraction, inconsistent activation, and signal variability. Evidence regarding the efficacy of training interventions targeting residual muscle activation in transtibial amputees (TTAs) to enhance prosthetic control remains limited. This study investigated whether a 4-week structured training could improve residual muscle control in TTAs.

**Methods:**

Nine male unilateral TTAs were assigned to either (A) biofeedback training with daily home exercises or (B) home exercises alone. Biofeedback sessions provided real-time surface electromyography (EMG) feedback on Tibialis Anterior, Gastrocnemius, and Peroneus Longus activity. Performance was evaluated at baseline and post-intervention using contraction accuracy (root mean square error), signal stability (time outside bounds), and selective muscle activation (co-contraction index).

**Results:**

Despite substantial inter-individual variability, participants demonstrated overall improvements in accuracy, stability, and selective muscle activation, irrespective of group. These findings suggest that consistent home-based training alone can induce rapid neuromuscular adaptation with measurable improvements in residual muscle control. However, this study was exploratory in nature and not powered to detect small between-group differences. Inter-individual variability highlighted the influence of baseline control and amputation etiology on outcomes, and one participant reported adverse neuropathic pain, underscoring the need to screening for neuropathic susceptibility, especially in individuals with prior neuroma or chronic phantom limb pain.

**Conclusions:**

Overall, structured EMG-based training improved residual muscle control in TTAs. These findings support the potential of EMG-guided interventions to foster more intuitive and reliable prosthetic use and to inform future rehabilitation protocols.

## Background

In recent years, a growing body of research has begun to challenge the notion that passive or autonomous control is sufficient for lower limb prostheses. Significant advancements have been made in neural and myoelectric control of prosthetic limbs, aiming to achieve seamless integration between user intention and prosthetic action and improve the overall quality of life for amputees by delivering more natural, responsive, and coordinated movements [1, 2]. However, compared to upper-limb systems, which have seen notable progress in direct muscle control, lower-limb prosthetics have lagged behind [3, 4]. This disparity can be attributed to complexity of lower-limb biomechanics. For instance, lower-limb controllers face stricter safety demands (terrain changes, mode transitions, stumble recovery) and often require user- and task-specific tuning, complicating robust generalization compared with upper-limb systems. Reliable electromyography (EMG) signal acquisition in the lower limb presents additional challenges compared with the upper limb. As the residual limb must bear weight during stance, surface EMG recordings are more susceptible to motion artifacts and pressure-induced changes in electrode-skin contact, which can compromise signal stability. Moreover, altered residual limb anatomy post-amputation and muscle reinnervation might contribute to atypical activation patterns [5], which can complicate the use of control signals.

One significant issue in effective implementation of myoelectric control is the co-contraction of residual limb muscles, where simultaneous activation of primary and non-primary muscles can complicate the use of EMG as a control signal [3, 4]. Research has shown that co-contraction of residual limb muscles is greater than in the intact limb and able-bodied controls across all phases of gait [6]. This increased co-contraction is generally interpreted as a compensatory strategy arising from reduced distal sensory feedback [6], the persistence of pre-amputation neural control strategies despite altered biomechanics [7], and stabilization of the limb–socket interface, particularly during swing. However, while these strategies may enhance perceived mechanical robustness, they reduce muscle selectivity and conflict with the requirements of EMG-based control, complicating the isolation of distinct and reliable control signals. Another significant challenge lies in achieving accurate and consistent contraction levels. The relationship between muscle activation and desired prosthetic output needs to be robust and repeatable. The difficulty in producing EMG signals within the desired range and signal fluctuations impair precise mapping between user intention and device action and may lead to unpredictable prosthetic responses or even safety hazards [8].

While reliable and selective EMG signals are essential for intuitive prosthetic control, residual muscle activation in transtibial amputees (TTAs) is often characterized by high co-contraction, instability, and poor repeatability. The development of adaptive control systems capable of handling EMG variability, co-contraction, and signal inconsistency is therefore necessary. However, the possibility that targeted user training could improve the quality of residual muscle signals themselves must also be considered. Previous research in upper-limb prosthetics has demonstrated the efficacy of biofeedback training in improving myoelectric control capabilities, enabling users to refine muscle activation patterns [9–11]. Such training supports improved residual limb awareness and facilitates the isolation and grading of muscle contractions.

Evidence from human motor control studies indicates that the neuromuscular system is capable of high-resolution voluntary control, even within muscle groups that are typically co-activated. For example, individuals can learn to differentially activate distinct muscle compartments in the upper limb when provided with appropriate feedback, demonstrating voluntary decoupling within synergistic muscles [12]. Recent work further suggests that similar dissociation of motor unit activity is possible in synergistic lower-limb muscles under feedback-guided conditions [13], supporting the physiological plausibility of refining selective control through training.

Whether similar training paradigms are effective for lower-limb amputees, however, remains an open question. Brief training has been shown to improve volitional activation of residual muscles in virtual myoelectric tasks [7, 14, 15], highlighting the neuromuscular system’s capacity for rapid adaptation. Moreover, a case study using direct EMG control of a powered ankle prosthesis demonstrated that structured training could enhance postural control and produce more synchronized activation between residual and intact limb muscles [16]. Studies have also reported that simply using a myoelectric lower‑limb prosthesis, without explicit EMG‑focused training, does not necessarily lead to measurable improvements in residual muscle activation [17]. This suggests that exposure to a myoelectric device alone is insufficient to drive meaningful neuromuscular adaptation.

In this context, biofeedback can be delivered through multiple sensory modalities, including visual, auditory, and haptic cues. Visual EMG feedback remains the most commonly used approach in myoelectric training research, as it provides high temporal resolution, intuitive mapping between muscle activation and output, and rich information content that supports early motor learning and the refinement of activation patterns. While alternative feedback modalities may offer advantages in specific contexts, particularly for use outside laboratory settings, visual feedback provides a well-established and controlled framework for investigating training-induced changes in residual muscle control. Although visual feedback itself is not intended as a long-term control interface, it is commonly used during training to facilitate motor learning and the development of more automatic, internally driven muscle control strategies that can later be expressed without external feedback.

Critically, it remains unclear whether structured training can consistently reduce co-contraction, improve signal accuracy and stability, and thereby address the limitations that currently hinder practical myoelectric control in lower-limb prostheses. Given considerable inter‑individual variability in residual muscle activation patterns in TTAs [18, 19], it is also unknown whether EMG-based training produces similar benefits across individuals, or whether some users may respond less robustly than others. In addition, the optimal approach to training, encompassing different methodologies and their relative impact on functional outcomes, warrants further investigation. It is also crucial to assess potential adverse effects associated with training regimens, such as muscle fatigue, discomfort, or the development of phantom limb pain, to ensure the safety and long-term adherence of participants.

This study aims to address key gaps in residual muscle control, including excessive co-contraction, inconsistent activation, and poor EMG signal stability, by investigating the efficacy of a comprehensive intervention incorporating both EMG-based biofeedback training and home exercises in enhancing residual muscle control in TTAs. Specifically, we seek to determine if structured training can improve the accuracy of muscle activation, reduce co-contraction, and lead to more stable and predictable control signals. By comparing a group performing only home exercises with a group receiving biofeedback training alongside the home exercises, we aim to shed light on the potential value of biofeedback during training. EMG-based biofeedback in this context refers to real-time visual feedback of surface EMG amplitude from residual limb muscles, with tasks structured to encourage participants to accurately match target activation levels and to selectively activate individual muscles while minimizing activity in non-target muscles. We hypothesized that such structured training would lead to measurable improvements in key aspects of myoelectric performance, namely activation accuracy, signal stability, and muscle selectivity. The findings of this research will provide valuable and clinically relevant insights into the design of effective rehabilitation protocols for lower limb amputees, aimed at addressing fundamental limitations that currently hinder intuitive and reliable EMG-based control of lower-limb prostheses.

## Methods

### Participants

Nine male unilateral TTAs participated in this study (mean ± SD, age = 50.8 ± 17.5 years; weight = 93.1 ± 13.8 kg; height = 179.8 ± 6.9 cm), described in Table 1. The National Bioethics Committee granted ethical approval for the study (protocol number: CII2024052847, approved 26.6.2024). All participants provided written informed consent prior to study enrollment.

**Table 1:** Participant demographics and clinical history

Subject	Reason of amputation	Age (years)	Height (cm)	Weight (kg)	Amputation side	Post-amputation (years)	Group
S01	Congenital	50	165	65	Right	50	A
S02	Trauma	64	180	104	Left	9	A
S03	Trauma	44	183	86	Left	27	A
S04	Neurofibromatosis	33	176	98	Right	26	B
S05	Trauma	67	186	82	Right	11	B
S06	Trauma	76	183	100	Left	13	B
S07	Trauma	21	181	100	Left	1	B
S08	Trauma	43	188	110	Left	20	B
S09	Trauma	59	176	100	Left	22	B

### Experimental design

Participants were assigned to one of two groups, with allocation determined by practical considerations such as availability during the workday and ability to travel for on-site training. Both groups underwent a baseline evaluation and a final evaluation after a 4-week intervention period. Group A (participants S01–S03) participated in six biofeedback training sessions over the course of four weeks, in addition to performing daily home exercises (excluding days with biofeedback sessions). Group B (participants S04–S09) performed only the daily home exercises throughout the 4-week period. The baseline and final evaluations, as well as all biofeedback training sessions (for group A), were identical in task structure, procedures, and setup.

During the first biofeedback session, the residual Tibialis Anterior (TA), Peroneus Longus (PL), and Gastrocnemius (GAS) muscles were identified using ultrasound (Terason uSmart-3200T-NexGen, Burlington, USA), as described in ref. [20]. Muscle bellies and fiber orientations, as well as nearby anatomical landmarks, were marked on a calf sleeve for consistent placement of EMG electrodes across sessions. The medial head of the GAS was used for all participants, except for participant S07, for whom the lateral head was selected due to stronger muscle activation on that side. Additionally, PL was excluded for S07, as both the muscle and the fibula had been removed during amputation surgery.

To monitor adherence and assess potential adverse effects, all participants were sent a weekly online questionnaire to complete, answering questions about the frequency of exercise performance as well as the presence of any pain, phantom pain or sensations, muscle cramps, or other training-related side effects.

### Biofeedback setup

**Fig. 1:**
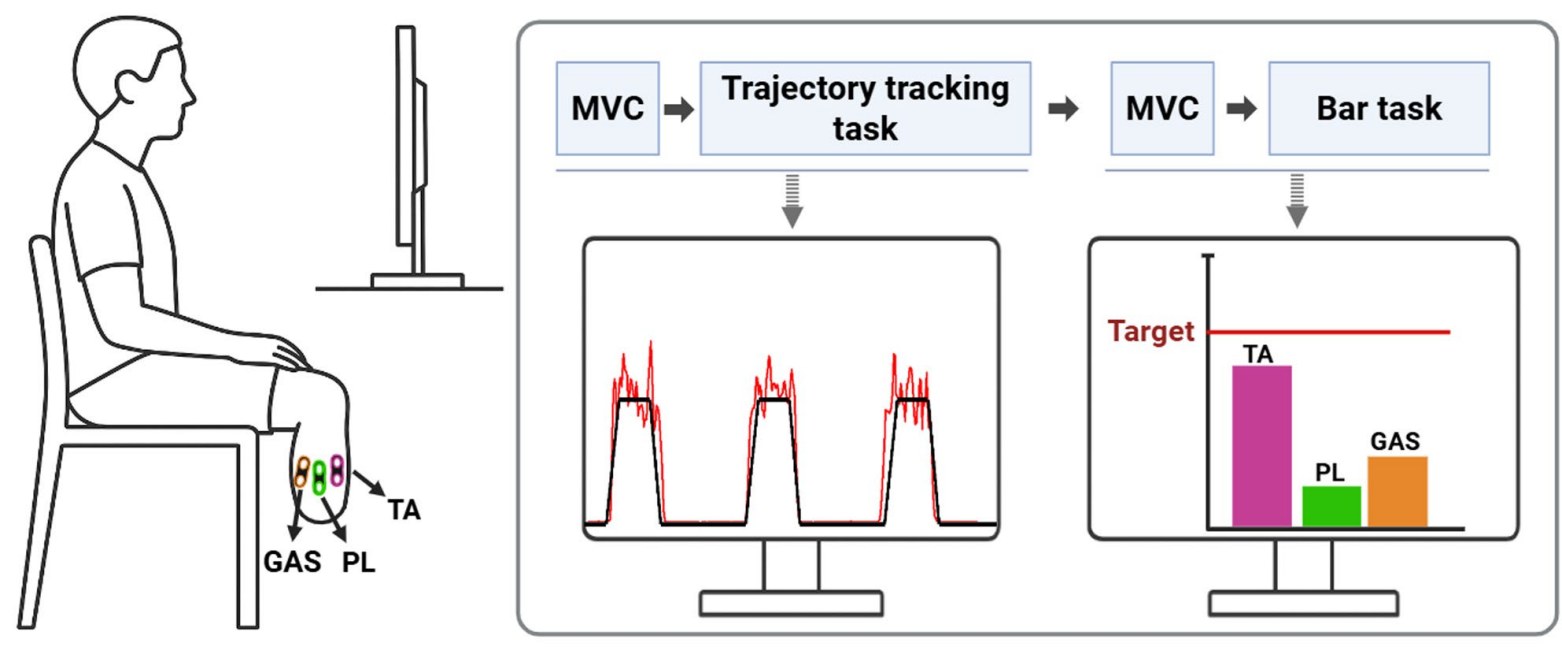
Experimental biofeedback setup. Wireless surface EMG electrodes placed on Tibialis Anterior (TA), Peroneus Longus (PL), and Gastrocnemius (GAS) recorded activity, which was displayed on a monitor as real-time visual feedback during trajectory tracking and bar tasks

Wireless surface EMG (sEMG) electrodes (Trigno™, Delsys Inc., USA), were used to collect EMG data. Prior to electrode placement, the skin was cleaned with alcohol wipes to reduce impedance and improve signal quality. Electrodes were placed on each of the identified residual limb muscle bellies and secured with a soft bandage to maintain stable contact. During sessions, participants sat in front of a monitor and received real-time visual feedback on their muscles’ activity (Fig. 1). During each experimental session, maximum voluntary contraction (MVC) of muscles was recorded separately for each task. The MVC was defined as the highest root mean square (RMS) value between two 3-second maximal voluntary attempts. An MVC was first recorded prior to the trajectory tracking task, followed by task execution. MVCs were then recorded again prior to the bar task to account for potential fatigue effects from the first task. Participants were allowed to rest between tasks as needed.

#### Trajectory tracking task: single-muscle feedback

Participants were then asked to follow a target trajectory using submaximal contractions of the selected muscle. The trajectory consisted of eight repetitions at 40% of the muscle’s MVC, each lasting 3 s, with 10-second rest intervals between contractions.

#### Bar task : multi-muscle feedback

Participants viewed a bar graph displaying real-time activation levels of all three muscles simultaneously. Participants were instructed to selectively activate one target muscle corresponding to specific ankle movements—dorsiflexion for TA, plantarflexion for GAS, and eversion for PL—while minimizing activation of the other two. For each activity, participants were asked to reach target activation levels of 20% (8 times), 40% (6 times), and 60% (3 times) of MVC. A 10-second rest period was provided between contractions.

### Home training exercises

After baseline evaluation, all participants, regardless of group allocation, received standardized instruction on how to perform the home exercises. The home exercise program focused on three specific ankle movements: dorsiflexion, plantarflexion, and eversion. Participants were instructed to contract the corresponding muscles to approximately 50% of their maximum effort. Each movement was repeated 10 times with 10 s of rest between contractions. These exercises were to be performed at least once per day and preferably using both legs simultaneously to facilitate bilateral motor awareness. Participants were guided to palpate the skin over the target muscle group during each exercise, with one hand placed posteriorly over the GAS and the other placed anteriorly and laterally to cover the TA and PL, to ensure correct muscle activation.

All participants received printed instructions with descriptions and figures illustrating the exercises. These materials provided guidance on identifying muscle locations using anatomical landmarks (e.g., tibia and fibular head), ankle movements, and reminders of which muscles should be felt contracting during each movement.

### Data acquisition and analysis

EMGworks Acquisition software (Delsys Inc., USA) was used to record EMG signals at a sampling rate of 2148 Hz during all biofeedback tasks. Signals were simultaneously processed to provide real-time visual feedback to participants, and all data were stored for subsequent offline analysis. To prepare the signals for real-time visualization, a 2nd-order Butterworth band-pass filter (20–450 Hz) was applied during acquisition, and an RMS envelope with a 250 ms window was used. For offline analysis, further processing was performed to ensure signal quality. This included a 2nd-order Butterworth high-pass filter at 25 Hz, a band stop filter with cutoff frequencies of 49–51 Hz to remove power line interference, a wavelet denoising approach [21] and an RMS envelope with a 250 ms window were applied. A 250 ms RMS window was selected to capture physiologically relevant fluctuations in muscle activation while providing sufficient smoothing for the assessment of EMG signal stability. Post-processing was performed in EMGworks Analysis (Delsys Inc., USA), MATLAB and R software.

For the trajectory tracking task, performance was quantified in terms of accuracy and signal stability. Accuracy was evaluated using root mean square error (RMSE) between the actual RMS values and the target intensity (40% of MVC). Signal stability was assessed using the time outside bound (TOB), defined as the percentage of time the RMS signal remained outside a ± 20% range around the mean value of each contraction, relative to the total recorded duration of that contraction.

For the bar task, selective muscle activation was quantified using co-contraction index (CCI). The CCI was calculated separately for each primary–non-primary muscle, using the normalized RMS amplitude of the primary and one non-primary muscle of each activity at a time:

$$ \:CCI = \frac{{EMG_{{non - primary}} }}{{\:EMG_{{primary}} }} * 100\: $$

Subsequently, the mean CCI for each contraction was determined. CCI = 0: perfect selectivity; CCI = 100: equal primary and non-primary activation; CCI > 100: non-primary dominates.

### Statistics

To examine the impact of the training programs on the outcome measures, linear mixed-effects models (LMMs) were used to consider repeated measurements within participants and unbalanced group sizes. The subject was included as a random intercept in all models to account for within-subject variability.

For measures related to trajectory tracking task performance, separate LMMs were constructed for RMSE and TOB. The fixed effects in these models included muscle (TA, PL, GAS), evaluation session (pre-training, post-training), and group (A, B). All two-way and three-way interactions among the fixed factors were also included. To evaluate changes in selective muscle activation in the bar task, an LMM was applied to the CCI. This model incorporated fixed effects of muscle (TA, PL, GAS), intensity of muscle activation (20%, 40%, 60%), activity (dorsiflexion, plantarflexion, eversion), evaluation session (pre-training, post-training), and group (A, B), as well as all possible interactions among these fixed factors.

Prior to model fitting, outcome variables were inspected for adherence to model assumptions. Because residual distributions deviated from normality, appropriate transformations were applied: log-transformation for RMSE, square-root transformation for TOB, and Box–Cox transformation for the CCI. Model assumptions were subsequently evaluated through visual inspection of Q–Q plots, as well as residuals-versus-fitted plots and formal tests of heteroscedasticity, which indicated no violations of homogeneity of variance.

Fixed effects and interactions were assessed using ANOVA derived from the fitted LMMs. Significant main effects and interactions were investigated further using post-hoc comparisons, performed on the LMMs with Tukey-adjusted correction for multiple comparisons. The significance level for all statistical tests was set at α = 0.05.

## Results

Group A (participants who received biofeedback training in addition to home exercises) demonstrated high adherence to the prescribed protocol, reporting an average of 7.7 (range 7–9) home-training sessions per week on non-biofeedback days. Group B (participants who performed only home exercises) averaged 4.9 (range 3–7) sessions per week. No significant correlations were found between training frequency and any outcome measure.

One participant in group B (S09) reported experiencing phantom and neuropathic pain during the first week of home training, which led to discontinuation of the training. Symptoms resolved within a week of cessation. Although a second evaluation was completed five weeks after baseline, data from this participant were excluded from further analysis due to incomplete training as prescribed. Another participant in group B (S04), who reported mild phantom pain prior to the training, noted no change in the intensity or frequency of pain throughout the training period. In addition, S08 experienced muscle cramps a few times during the training period and S07 reported pricking sensations in the residual limb after final evaluation and a few times after home training. No other participants reported pain, discomfort, muscle cramps, or other training-related adverse effects.

### Accuracy of muscle activation (RMSE)

**Fig. 2:**
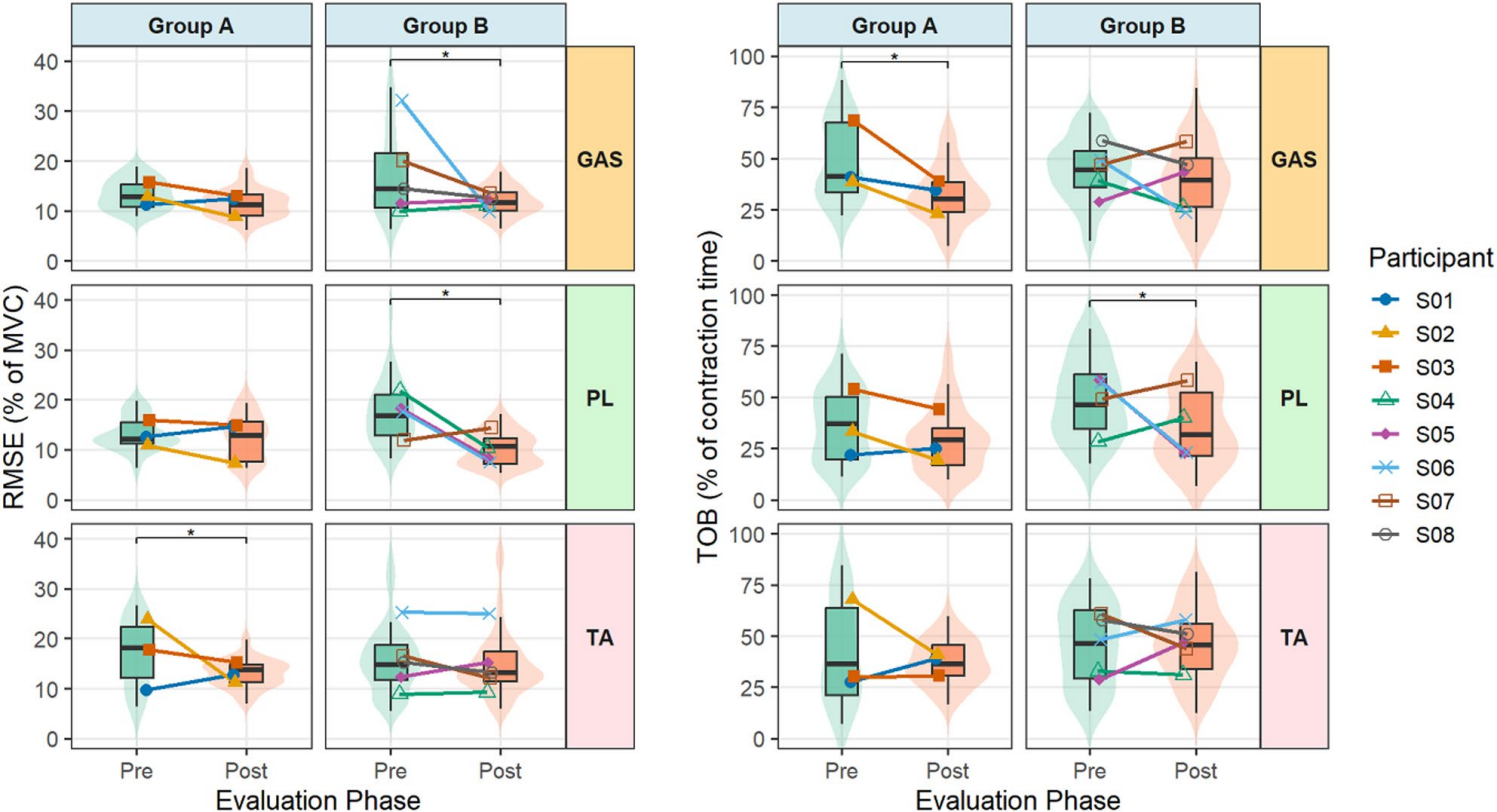
Accuracy (Root mean square error, RMSE, left), and signal stability (time outside bounds, TOB; % of contraction time outside ± 20% of mean RMS, right) during trajectory tracking task pre- and post-training. Results are shown separately for muscles (TA, tibialis anterior; PL, peroneus longus; GAS, gastrocnemius) and groups (A: biofeedback + home training; B: home training only). Boxplots show median ± IQR with violin distribution of all contractions across all participants; colored points denote each participant’s mean, connected across sessions. * *p* < 0.05

The analysis of data from the trajectory tracking task revealed no main effect of group (*p* = 0.7). Across both group A and B, mean accuracy improved post-training, although individual responses varied. A main effect of muscle was also observed (*p* < 0.006), indicating differences in accuracy between GAS, PL, and TA. A 3-way interaction was found between muscle, group, and evaluation session, reflecting slight group differences regarding the magnitude of change in pre- to post-training RMSE values across the three muscles (*p* = 0.003; Fig. 2). Across participants, the mean pre– to post change in RMSE was − 4.3 ± 7.8% for GAS, − 4.5 ± 6.0% for PL, and − 2.0 ± 5.1% for TA.

### Signal stability (TOB)

Stability of muscle activation during the trajectory tracking task generally improved. An overall reduction in TOB was seen from pre- to post-training (*p* < 0.001) with no significant difference between groups (*p* = 0.35). However, muscle-specific differences were evident in response to training (muscle * session interaction; *p* = 0.03, Fig. 2). GAS and PL showed significant reductions in TOB, with mean pre– to post changes of − 9.6 ± 7.8% (*p* < 0.001) and − 10.0 ± 6.0% (*p* = 0.001), respectively, whereas TA showed negligible change (− 1.7 ± 5.1%, *p* = 0.78).

### Co-contraction (CCI)

**Fig. 3:**
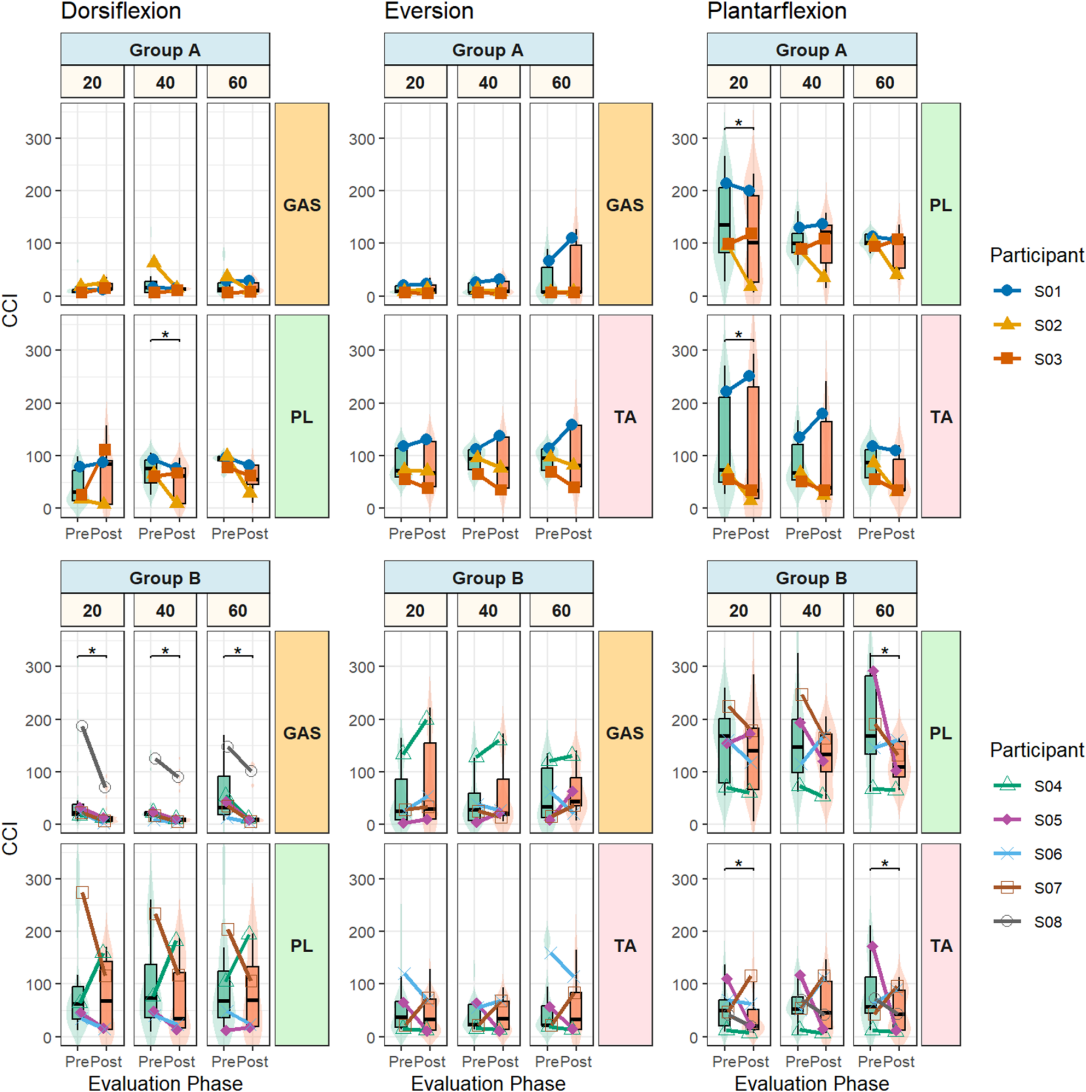
Selective muscle activation during bar task quantified using the co-contraction index (CCI) pre- and post-training. Results are shown for muscles (TA, tibialis anterior; PL, peroneus longus; GAS, gastrocnemius), groups (A: biofeedback + home training; B: home training only), activities (dorsiflexion, plantarflexion, eversion) and intensities (20/40/60% MVC). Boxplots show median ± IQR with violin distributions of all contractions across participants; colored points denote each participant’s mean, connected across sessions. * *p* < 0.05. CCI: 0 = perfect selectivity; 100 = equal activation; >100 = non-primary dominance

Analysis of the CCI revealed that, despite substantial inter-individual variability, overall improvements in selective muscle activation were observed at group-level following training (*p* < 0.001), corresponding to a shift toward lower CCI values (i.e., reduced relative activation of non-primary muscles). Main effects of muscle (*p* < 0.001), activity (*p* < 0.001), and intensity (*p* = 0.005) were also observed, with no main effect of group (*p* = 0.65). However, several higher-order interactions were significant, including muscle × activity (*p* < 0.001), activity × session (*p* < 0.001), muscle × group (*p* < 0.001), muscle × activity × group (*p* = 0.014), and muscle × activity × session × group (*p* = 0.049), indicating that the effect of training on selective activation was activity- and muscle-dependent, and that these dependencies differed between groups (Fig. 3).

Post-hoc comparisons showed that during dorsiflexion, CCI significantly decreased following training with mean reductions of − 18.6 ± 23.5% for GAS (*p* < 0.0001) and − 11.1 ± 70.6% for PL (*p* = 0.012). During plantarflexion, CCI also decreased significantly, by − 25.7 ± 38.3% for PL (*p* = 0.002) and − 11.5 ± 53.8% for TA (*p* < 0.001). In contrast, during eversion, CCI decreased slightly for TA (− 5.7 ± 34.2%, *p* = 0.20) but increased for GAS (+ 10.5 ± 17.0%, *p* = 0.10). Post-hoc comparisons further revealed that, aside from PL during dorsiflexion in group A (*p* = 0.02), no significant differences in CCI were observed between contraction intensities.

## Discussion

The aim of this study was to examine whether a structured residual muscle training program could improve residual muscle control in TTAs across three key domains relevant to myoelectric prosthetic control: activation accuracy, signal stability, and selective muscle activation. We hypothesized that structured training would lead to measurable improvements in these domains, and our results partially supported this hypothesis, with group-level improvements observed across all three measures despite substantial inter-individual variability. Contraction accuracy generally improved from pre- to post-training in all muscles. Stability of the activation signal also showed improvement, mainly in GAS and PL. Finally, selective activation improved, with lower co-contraction for dorsiflexion and plantarflexion, although the interactions may point to the complexity of being selective by lowering activation of non-primary muscles. Together, these parallel improvements in accuracy, consistency, and selectivity suggest that even a relatively brief, focused training regimen can foster rapid neuromuscular adaptations in TTAs, enhancing their ability to produce controlled, repeatable EMG commands.

Although group A received structured biofeedback sessions in addition to home exercises, their gains did not exceed those of group B in any metric. In addition to the effects of a small sample size, the absence of between-group differences likely reflects that the common elements of daily training sessions were already potent drivers of early adaptation. Similar improvements from home training have also been demonstrated in upper-limb amputees, where extended home use led to measurable gains in myoelectric control and functional performance [22]. Although our results suggest that even unsupervised home training can lead to measurable gains in volitional control, it is important to consider that the biofeedback dose was modest (six sessions in four weeks) and not explicitly progressive or individualized, and that biofeedback training may have produced benefits in aspects of control that were not measured, such as faster stabilization and reduced effort.

Although overall gains were demonstrated in accuracy, stability, and selectivity, marked inter-individual variability was seen and can, in part, be attributed to the etiology and developmental history of the amputation. Participant S01, the only congenital TTA, showed no improvement across outcomes despite biofeedback and consistent home training. Congenital limb deficiency is said to lead to lifelong neural and proprioceptive adaptations that differ from those of acquired amputees [23] and may require alternative or more intensive training approaches. Variations in residual limb length, muscle mass, and surgical approach might also change the richness of afferent feedback and effectiveness of training. Baseline task performance also influenced training response. Participants with high baseline errors tended to show greater improvement. In contrast, those starting with relatively low errors had less improvement, plateaued, or even regressed slightly. Initial task performance must therefore be considered to allow realistic expectations regarding response and benefits of training programs, and to be able to provide personalized training protocols, through adjustment of feedback intensity, modality, or task difficulty to promote consistent progression.

### Single-muscle control (trajectory tracking)

Our single-muscle trajectory tracking at 40% MVC showed significant improvement, with post-training RMSEs of ~ 11–14% for GAS, PL, and TA (Fig. 2). These values are slightly higher than those reported by Rubin et al. [5], where RMSE values were ~ 2–7% for TA and GAS when tracking at 20–35% MVC. This may, in part, stem from differences in parameters such as target intensity, task design, and analysis metrics, that can influence these measures of tracking accuracy. Using performance metrics other than RMSE prevents direct numeric comparisons but the studies still show similar trends. Alcaide-Aguirre et al. [14] reported sizable early tracking errors in TTAs that improved from being 33% (static) and 40% (dynamic) larger than controls to non-significant after brief training. Although they did not report absolute %MVC-normalized RMSE values, the pattern closely mirrors our pre- to post-training improvement in accuracy. Similarly, Fleming et al. [15] demonstrated that TTAs can adapt residual antagonistic muscle control in a dynamic context. Using proportional EMG from residual TA and GAS to balance a virtual inverted pendulum, participants progressively reduced falls and sway. Although the specific task demands differed, the consistent improvements across studies reinforce the broader interpretation that residual-muscle control in TTAs is trainable across diverse contexts.

In addition to subject variability, a high variability between muscles across participants was seen. GAS and PL generally showed greater improvements, while TA showed more modest changes. One possible explanation is that the TA often undergoes greater atrophy post-amputation than GAS [19], which may limit its capacity to generate strong or consistent contractions and thereby constrain training-related gains.

Given the small and unbalanced sample sizes, muscle- and group-specific differences in the magnitude of improvement should be interpreted cautiously, as they are likely influenced by inter-individual variability and baseline performance rather than reflecting systematic effects of the training protocols.

### Multi-muscle control (co-contraction)

In the multi-muscle task, co-contraction generally decreased for antagonists during dorsiflexion and plantarflexion, whereas eversion showed minimal or no improvement. Previous work has shown that even with agonist–antagonist myoneural interface (AMI) reconstruction, individuals control dorsiflexion and plantarflexion more naturally and robustly than eversion and inversion. This disparity is markedly greater in non-AMI amputees and is reflected in significantly lower motor controllability scores for eversion/inversion compared to dorsiflexion/plantarflexion [24, 25]. In non-AMI amputees, participants might revert to habitual synergies, such as a plantarflexion–eversion coupling, leading to spillover activation of GAS, a pattern not typically observed in intact or AMI residual limbs [24]. This interpretation aligns with the upward trend in GAS co-contraction during eversion observed in our data. Moreover, during eversion, TA must be intentionally suppressed to avoid interfering with the eversion, as it naturally acts as a dorsiflexor–invertor. The observed co-contraction suggests that the modest training dose in this study may not have been sufficient to improve this selective inhibition.

The lack of intensity effects on co-contraction (20/40/60% MVC) indicates that the ability to suppress non-primary muscle activation was not dependent on the target contraction amplitude within the submaximal range. Because both primary and non-primary muscle EMG amplitudes were first normalized to their respective MVC values before computing the CCI, the metric reflects the relative proportion of each muscle’s activation capacity, rather than absolute signal magnitude. Under this normalization, a proportional increase in activation of both the primary and any co-activated muscles across intensities would yield a similar co-contraction. Thus, participants who maintained a consistent activation pattern, whether optimal or suboptimal, across target amplitudes would present comparable co-contraction values at 20%, 40%, and 60% MVC. This suggests that the training influenced the pattern of muscle recruitment in a way that generalized across tested intensities, rather than selectively improving performance at a particular target level.

Prior work has established that brief practice and visual feedback can modify residual activation patterns in TTAs, supporting trainability of antagonist coordination [15, 26]. However, to our knowledge, no controlled study has quantified training-induced changes in residual muscle co-contraction in TTAs using a CCI metric. Studies on individuals with an AMI amputation report markedly reduced unintended co-contraction compared to non-AMI amputations through surgical restoration of agonist–antagonist coupling [24, 25], offering an upper bound on what improved selectivity can look like. Although these findings are promising for myoelectric control of prosthetics, they represent surgical intervention, as opposed to a non-invasive approach such as training. The present results extend the literature by providing quantitative evidence that training alone can reduce co-contraction in specific muscle–activity combinations, while also revealing contexts, such as eversion, where improvements remain limited. TTAs lack internal proprioceptive feedback from muscle spindles and Golgi tendon organs that typically guides fine-tuned agonist–antagonist coordination in the intact limb, which limits the ability to optimize selective control based solely on intrinsic sensory cues. In our study, external feedback consisted primarily of visual feedback, and for home training, participants were encouraged to touch the skin over the target muscle to enhance awareness of muscle activation. Prior studies have successfully demonstrated that augmented visual [9], auditory [27], and haptic feedback [28, 29] can enhance residual muscle control by providing real-time cues that help compensate for missing proprioception.

From a control systems perspective, systematic training may enhance the robustness of EMG inputs by increasing signal separability, making activation patterns more distinct across muscles and reducing unintended cross-talk between channels. This eases the demands on pattern recognition or regression algorithms, leading to more accurate decoding of user intent [30, 31]. When such separability remains suboptimal, various algorithmic control strategies have been proposed to maintain control accuracy, such as adaptive weighting that dynamically adjusts the contribution of EMG signals [32, 33] and exploitation approaches that leverage and adapt to co-contractions rather than trying to eliminate them [34]. Multimodal input fusion may also be feasible, such as combining EMG with inertial sensors, foot-switch timing, or even visual cues, which enable prosthesis control systems to infer user intent more reliably by cross-validating signals across complementary modalities [35, 36]. Integrating such adaptive or multimodal control schemes with user training may yield the most resilient EMG-driven systems.

### Clinical and practical considerations

Adherence to rehabilitation protocols and consistent engagement can influence motor re-education and residual limb function in TTAs [37]. In this study, most participants in both groups trained close to the recommended daily frequency; however, adherence in Group B was more variable, with some individuals completing substantially fewer sessions. Despite this variability, no significant correlations were found between training frequency and outcome measures, likely reflecting the limited sample size. This suggests that within the range of adherence observed, training frequency alone did not account for improvements in control. We did not predefine a compliance threshold, as is sometimes applied in rehabilitation trials, but future studies with larger cohorts may benefit from stratifying outcomes based on such criteria to better capture potential dose–response effects.

The structured nature of biofeedback sessions and regular contact with the research team may have contributed to slightly greater adherence of group A compared to the home-exercise-only group, where some participants performed exercises less frequently than recommended. This highlights a common challenge in self-directed rehabilitation programs, which are less resource-intensive but may suffer from inconsistent execution. Emerging strategies such as mobile applications, wearable sensors and gamified training platforms could deliver real-time feedback remotely, supporting adherence while reducing the need for in-person supervision. In addition, alternative forms of feedback beyond visual displays, such as vibrotactile or auditory cues, may provide practical options for integration into daily life, where lab-based visual setups are less feasible. Exploring these modalities, as well as adaptive feedback intensity or personalized progress tracking, may enhance both adherence and training effectiveness in future rehabilitation programs.

Day-to-day variability is also a significant and often underestimated confounder that likely contributed to the heterogeneity we observed. We noted that participants in group A did not always perform consistently across the different biofeedback training sessions. Although this intra-individual variability was not formally reported in the results section, it was evident during training. Residual limb volume fluctuations, due to fluid shifts and socket doffing, are substantial in lower‑limb prosthesis users, and studies have documented notable volume changes between morning and afternoon sessions [38, 39]. Such fluctuations can affect electrode spatial positioning even with ultrasound‑guided placement and marking sleeves, which may perturb the recorded motor-unit populations and affect EMG features [40], particularly amplitude‑based metrics. In addition, even with consistent alcohol-based skin preparation, day-to-day changes in skin condition and electrode–skin impedance at the residual limb may have introduced additional variability in amplitude-based EMG measures. Beyond peripheral factors, neuromuscular performance can exhibit time‑of‑day variability. Prior research demonstrates that MVC strength and neuromuscular coordination often vary diurnally [41]. Although the evidence is less clear for EMG steadiness or fatigability metrics, such rhythmic influences on force production could nonetheless impact MVC normalization and submaximal control consistency across sessions.

In this study, EMG signals were sampled at 2148 Hz, higher than the 1000 Hz commonly used in prosthetic and myoelectric control research, to provide a wider guard band beyond the ~ 450–500 Hz EMG bandwidth [42], minimize aliasing, and enhance temporal precision for analyses such as wavelet denoising and RMS envelope estimation. Methodological parameters including sampling rate, filtering, and RMS window length can influence signal smoothness and apparent performance; while control-oriented pipelines often apply stronger smoothing to stabilize commands, our milder approach aimed to reflect the underlying physiology more faithfully. Because both pre- and post-training sessions used identical acquisition and processing pipelines, relative performance changes remain unbiased despite potential differences in absolute values compared to other methods. The design of the real-time biofeedback system, such as update rate, filtering, and latency, was also critical in shaping user experience: the chosen parameters yielded smooth yet responsive feedback that likely enhanced participants’ ability to interpret and modulate muscle activity. In clinical contexts, balancing feedback clarity, responsiveness, and immediacy is essential, as excessive filtering or latency can obscure subtle cues or weaken the perceived connection between effort and outcome, thereby limiting motor learning and rehabilitation efficacy.

### Adverse events

Adverse events were infrequent but clinically relevant. One participant (S09) with a history of neuroma treated 20 years ago, reported a recurrence of phantom and neuropathic pain, described as intermittent electric/tingling sensations, aggravated by contractions and worse at night, which led to early withdrawal. Such symptoms are consistent with known post-amputation pain mechanisms, particularly neuroma-related neuropathic pain, which is sensitive to stimulation [43]. By contrast, another participant (S04) with stable, pre-existing mild phantom pain, reported no change during training, suggesting that chronic, non-fluctuating phantom pain may not be exacerbated by low-intensity EMG-based interventions. The key difference between these two cases may lie in underlying pathology: S09 had a prior neuroma history, which can produce mechanically triggered neuropathic flares, whereas S04’s pain was stable and likely less sensitive to movement-induced peripheral inputs. Two other participants reported transient sensations such as muscle cramps and pricking which are common in amputees and often unrelated to serious pathology [44]. Overall, the 4-week program was well tolerated; however, individualized risk assessment prior to initiating residual limb muscle training, particularly in patients with a history of neuroma or chronic phantom limb pain, is necessary and training protocols might benefit from early screening for neuropathic susceptibility and close monitoring during initial training phases.

### Limitations

This study has several limitations. The small sample size, with all participants being male, limits both statistical power and the generalizability of our findings, particularly to female TTAs. The small and unequal group sizes further reduce sensitivity to detect between-group differences. The absence of statistically significant group effects should therefore be interpreted with caution and does not imply equivalence between training approaches. Although our participants varied in post-amputation time, cause of amputation, and pain history, the limited sample prevented meaningful exploration of how these factors influenced training responsiveness.

The absence of long-term follow-up also means we cannot determine whether the improvements persisted beyond the 4-week training. While the low-dose program, generic practice, and intrinsic feedback were sufficient to yield early gains, future studies should test the true added value of supervised biofeedback by exploring different dosing strategies, incorporating progressive and individualized task adjustments based on baseline deficits, and integrating real-time external feedback into daily home exercises to enhance engagement and outcomes.

## Conclusions

This study provides evidence that TTAs can achieve meaningful improvements in residual muscle control through structured training. Across participants, training enhanced contraction accuracy, signal stability, and selective activation, demonstrating the neuromuscular system’s capacity for rapid adaptation even years after amputation. Importantly, these gains were observed in both groups, indicating that consistent home-based training alone can foster measurable progress, while biofeedback may provide added structure and engagement without clearly superior outcomes in this small sample. The marked variability in individual responses highlights the need for personalized approaches to training, taking into account baseline control ability, and amputation etiology. Furthermore, the occurrence of adverse effects in one participant underscores the importance of screening for neuropathic pain risk before implementing residual muscle training. Overall, these findings provide promising evidence that targeted EMG-based training interventions can strengthen the foundations for more intuitive and reliable myoelectric prosthetic control.

## Data Availability

The datasets used and/or analysed during the current study are available from the corresponding author on reasonable request.

## Abbreviations

TA: Tibialis Anterior
PL: Peroneus Longus
GAS: Gastrocnemius
TTA: Transtibial amputee
EMG: Electromyography
sEMG: Surface Electromyography
MVC: Maximum Voluntary Contraction
RMS: Root Mean Square
RMSE: Root Mean Square Error
TOB: Time Outside Bound
CCI: Co-contraction Index
AMI: Agonist–Antagonist Myoneural Interface
LMM: Linear Mixed-effects Model
ANOVA: Analysis of Variance
